# Structural dynamics effects on the electronic predissociation of alkyl iodides

**DOI:** 10.1038/s41598-020-62982-0

**Published:** 2020-04-21

**Authors:** Marta L. Murillo-Sánchez, Alexandre Zanchet, Sonia Marggi Poullain, Jesús González-Vázquez, Luis Bañares

**Affiliations:** 1 Departamento de Química Física (Unidad Asociada I+D+i al CSIC), Facultad de Ciencias Químicas, Universidad Complutense de Madrid, 28040 Madrid, Spain; 2 Departamento de Química Física, Facultad de Ciencias Químicas, Universidad de Salamanca, 37003 Salamanca, Spain; 3 Instituto de Física Fundamental (IFF-CSIC), Consejo Superior de Investigaciones Científicas, Serrano 123, 28006 Madrid, Spain; 4 Department of Chemistry, University of California, Berkeley, California 94720 United States; 5 Departamento de Química, Módulo 13, Facultad de Ciencias, Universidad Autónoma de Madrid, 28049 Madrid, Spain; 6 Institute for Advanced Research in Chemical Sciences (IAdChem), Facultad de Ciencias, Universidad Autónoma de Madrid, 28049 Madrid, Spain; 7 Instituto Madrileño de Estudios Avanzados en Nanociencia (IMDEA-Nanoscience), Cantoblanco, 28049 Madrid, Spain

**Keywords:** Reaction kinetics and dynamics, Atomic and molecular interactions with photons

## Abstract

The correlation between chemical structure and predissociation dynamics has been evaluated for a series of linear and branched alkyl iodides with increasing structural complexity by means of femtosecond time-resolved velocity map imaging experiments following excitation on the second absorption band (*B*-band) at around 201 nm. The time-resolved images for the iodine fragment are reported and analyzed in order to extract electronic predissociation lifetimes and the temporal evolution of the anisotropy while the experimental results are supported by *ab initio* calculations of the potential energy curves as a function of the C-I distance. Remarkable similarities are observed for all molecules consistent with a major predissociation of the initially populated bound Rydberg states 6*A*″ and 7*A*′ through a crossing with the purely repulsive states 7*A*″, 8*A*′ and 8*A*″ leading to a major R + I*(^2^*P*_1/2_) (R = CH_3_, C_2_H_5_, *n*-C_3_H_7_, *n*-C_4_H_9_, *i*-C_3_H_7_ and *t*-C_4_H_9_) dissociation channel. The reported electronic predissociation lifetimes are found to decrease for an increasing size of the linear radical, reflecting the shifts observed in the position of the crossings in the potential energy curves, and very likely a greater non-adiabatic coupling between the initially populated Rydberg states and the repulsive states leading to dissociation induced by other coordinates associated to key vibrational normal modes. The loss of anisotropy is fully accounted for by the parent molecular rotation during predissociation and the rotational temperature of the parent molecule in the molecular beam is reasonably derived.

## Introduction

Time-resolved femtosecond pump-probe experiments supported by *ab initio* calculations have been largely reported in recent years for a variety of molecular systems, aiming at a deeper understanding of the nuclear and electronic dynamics occurring in different photodissociation and photoionization processes^1^. Among them, the correlation between increasing structural complexity of alkyl iodides and the ultrafast C-I bond cleavage was investigated by our group employing femtosecond time-resolved velocity map imaging (VMI)^2^ in conjunction with *ab initio* full-dimension time-resolved dynamics calculations. For this purpose, a series of linear and branched alkyl iodide molecules were excited at 266 nm in the first absorption band (*A*-band) which arises from the n(5p,I) → σ^*^(C—I) transition^3^. Several repulsive electronic states can be populated leading to dissociation mediated by a conical intersection either into the major channel R + I^*^(^2^*P*_1/2_) or into the minor one R + I(^2^*P*_3/2_) - henceforth denoted as I^*^ and I, respectively. Reported reaction times for both channels I^*^ and I showed to steadily increase with increasing R radical size. The experimental and theoretical findings were rationalized by a 1-D model correlating the reaction time *τ*, the reduced mass of the molecule and an energy variable, E’ = E_*av*_-E_*int*_, accounting for the difference between the total available energy, E_*av*_, and the internal energy of the radical R, E_*int*_^2^. The major role of the energy flux into the internal degrees of freedom of the molecule, vibration and rotation, was in particular demonstrated. This work was later extended to the halogen atom substitution in methyl iodide studying CH_2_ICl and CH_2_BrI photodissociation in the first absorption band^4^.

The goal here is to investigate the effect of key structural changes on the photodynamics of a prototypical electronic predissociation reaction. For this purpose, we study the time-resolved predissociation of a series of linear (CH_3_I, C_2_H_5_I, *n*-C_3_H_7_I, *n*-C_4_H_9_I) and branched (*i*-C_3_H_7_I, *t*-C_4_H_9_I) alkyl iodides in the second absorption band, labeled *B*-band, following excitation around 201 nm. The experimental results are complemented by *ab initio* calculations and compared to the well-known methyl iodide^5–9^.

The *B*-band results from the excitation of a non-bonding 5*pπ* electron of the iodine atom to a 6*s* molecular Rydberg orbital. In *C*_3*v*_ symmetry (*i.e*. for CH_3_I^10^ and *t*-C_4_H_9_I), five Rydberg states emerge from the coupling between the electron and the resulting ionic core, either ^2^*E*_3/2_ or ^2^*E*_1/2_ because of the spin-orbit interaction. The *B*-band is composed of the two states ^3^*E*_2_ and ^3^*E*_1_ -referred to as ^3^*R*_2_ and ^3^*E*_1_, respectively- arising from the ^2^*E*_3/2_ ionic core although the ^3^*R*_2_ ← $\tilde{X}$ transition is forbidden by symmetry (ΔΩ selection rule). The three additional states associated with the ^2^*E*_1/2_ ionic core constitute the third absorption band, *C*-band. In CH_3_I, the absorption spectrum of the *B*-band is dominated by the ${0}_{0}^{0}$ transition into the ^3^*R*_1_ in its vibrationally ground state and presents a well resolved vibronic structure including weak transitions involving *e.g*. the *v*_2_, *v*_3_, *v*_5_, and *v*_6_ vibrational modes^10^. In *C*_*s*_ symmetry (*i.e*. for C_2_H_5_I, *n*-C_3_H_7_I, *n*-C_4_H_9_I and *i*-C_3_H_7_I), the ^3^*R*_2_ and ^3^*R*_1_ states, which are *E* states in *C*_3*v*_, split each into two states, of *A*′ and *A*″ symmetry. The [5*A*″, 6*A*′] states, corresponding to the ^3^*R*_2_, are similarly dark states while the [6*A*″, 7*A*′] states, associated with the ^3^*R*_1_, are optically active and can be populated after excitation at around 201 nm.

Following excitation of CH_3_I at ~201 nm, an electronic predissociation through the coupling between the initially populated ^3^*R*_1_ and the repulsive ^3^*A*_1_ state leads to the mayor dissociation into CH_3_ + I*. A small amount of iodine in its ground spin-orbit state in correlation with CH_3_ remarkably ro-vibrationally excited was detected and attributed to a second curve crossing between ^3^*R*_1_ and the repulsive state ^1^*Q*_1_^9,11,12^. The corresponding quantum yield Φ^*^, defined as Φ^*^ = [I^*^]/([I^*^] + [I]), was found to range between 0.99 in the ${0}_{0}^{0}$ and 0.87 in the ${3}_{0}^{1}$ vibronic band, *i.e*. excitation in the C-I stretching mode (*v*_3_)^12^. Employing femtosecond VMI, a predissociation lifetime of 1.52 ± 0.10 ps was measured for the CH_3_ + I* channel^5^. The predissociation dynamics following excitation to vibronic bands was also investigated. The umbrella mode (*v*_2_) was found to enhance the coupling leading to a twice faster predissociation lifetime in the ${2}_{0}^{1}$ band while four times longer I* rising times were reported in the ${3}_{0}^{1}$ band following excitation in the C-I stretching mode (*v*_3_)^9^. The evolution of the anisotropy as a function of the pump-probe delay was recently analyzed in detail by means of a theoretical fitting procedure using quasi-classical theory. The role of methyl fragment angular momentum alignment was highlighted along with the bending motion and rotation of the parent molecule and methyl fragment^13^.

In contrast, alkyl iodide photodissociation dynamics beyond methyl iodide, has been little researched following excitation in the *B*-band. We recently investigated the photodynamics and stereodynamics of ethyl iodide at the origin of the *B*-band, employing nanosecond VMI in combination with high-level *ab initio* calculations of the potential energy curves as a function of the C-I distance^14^. A similar predissociation dynamics was reported characterized by a major formation of I* through the coupling between the initially populated Rydberg states, 6*A*″ and 7*A*′, and the corresponding repulsive states, labeled 7*A*″, 8*A*′ and 8*A*″. The minor C_2_H_5_ + I channel, characterized by Φ^*^ ≥ 0.9, was also observed and similarly attributed to a second coupling with the 4*A*″ and 5*A*′ states −*i.e*. ^1^*Q*_1_ in *C*_3*v*_− at shorter C-I distances. Femtosecond time-resolved VMI experiments were recently performed following excitation at 201.19 and 200.08 nm, corresponding to the ${0}_{0}^{0}$ origin and the ${18}_{0}^{1}$ transition, *i.e*. excitation in the *v*_18_ = 1 methyl torsion mode^15^. A predissociation lifetime of 1.34 ± 0.05 ps was reported for the C_2_H_5_ + I* following excitation in the origin of the band while the methyl torsion mode was found to enhance the coupling leading to a faster predissociation. The shorter lifetime at the ${0}_{0}^{0}$ in comparison with methyl iodide was attributed to a stronger coupling of the Rydberg state and the repulsive states. The evolution of the anisotropy as a function of the pump-probe delay was analyzed and attributed to the rotation of the parent molecule during dissociation, reflecting a rotational temperature in the molecular beam of 100 K.

**Figure 1:**
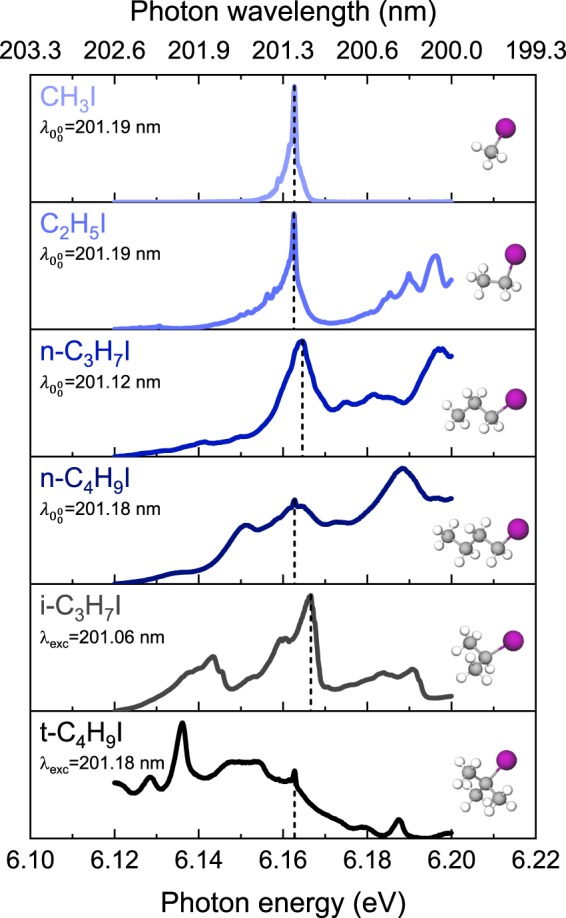
Absorption spectra of the series of linear (CH_3_I, C_2_H_5_I, *n*-C_3_H_7_I, *n*-C_4_H_9_I) and branched (*i*-C_3_H_7_I, *t*-C_4_H_9_I) alkyl iodides investigated in the present work (adapted from refs. ^16,17^). The excitation wavelength employed is indicated in each panel and highlighted by a vertical dashed line. For the linear molecules, this wavelength, labeled ${\lambda }_{{0}_{0}^{0}}$, correspond to the ${0}_{0}^{0}$ origin of the *B*-band^16^. For the branched alkyl iodides, *i*-C_3_H_7_I, *t*-C_4_H_9_I, the wavelengths used correspond to specific vibronic transitions, since the laser cannot be tuned to the corresponding ${0}_{0}^{0}$ transitions which are well shifted to the red.

In the present work, we have performed femtosecond time-resolved VMI experiments on a series of linear (CH_3_I, C_2_H_5_I, *n*-C_3_H_7_I, *n*-C_4_H_9_I) and branched (*i*-C_3_H_7_I, *t*-C_4_H_9_I) alkyl iodides excited in the *B*-band around ~201 nm. The absorption spectra for all molecules are reported in Fig. 1 (adapted from refs. ^16,17^) where the specific wavelength selected to excite each molecule is also indicated. The excitation wavelength for the linear molecules is assigned to the ${0}_{0}^{0}$ transition of the *B*-band, according to the early work of Boschi *et al*.^16^. In contrast, for the branched *i*-C_3_H_7_I, the selected wavelength is tentatively attributed to the vibronic band associated with the C-I stretching mode (or alternatively to the ${0}_{0}^{0}$ transition of the *C*-state)^17^, while for *t*-C_4_H_9_I, it is assigned to the ${6}_{0}^{1}$ vibronic transition of the *B*-state, where the molecule is excited in the *v*_6_ = 1 C-C stretching mode (806 cm^−1^ in the ground state)^18^.

The experimental and theoretical methodologies are presented in the next section while the most relevant results of the study are depicted and discussed in Section III. The translational energy distributions and the measured I* transients are reported for the series of linear (CH_3_I, C_2_H_5_I, *n*-C_3_H_7_I, *n*-C_4_H_9_I) and branched (*i*-C_3_H_7_I, *t*-C_4_H_9_I) alkyl iodides. In addition, the evolution of the anisotropy as a function of the pump-probe time delay for I* fragments is discussed in terms of the parent molecule rotational temperature. Finally, based on the results from high-level *ab initio* calculations the predissociation mechanism is discussed in terms of structural effects on the predissociation reaction.

## Methods

### Experimental method

The experimental setup has been described in detail in previous works^5,9,15^ and only the details relevant to the present experiments will be given here. A femtosecond chirped pulse amplified (CPA) Ti:sapphire laser system with a tunable central wavelength delivering 3.5 mJ laser pulses around 804 nm of 50 fs duration at 1 kHz repetition rate is employed. Around 1 mJ is used to pump a two-stage femtosecond automated optical parametric amplifier (TOPAS Prime Spectra Physics) whose output signal is two-step frequency quadrupled by means of two BBO crystals yielding tunable pulses centered around 304.5 nm with energies typically ~7–9 *μ*J and with a full width at half maximum (FWHM) bandwidth of ≈1.7 nm, to probe simultanously the I(^2^*P*_3/2_) and I*(^2^*P*_1/2_) atoms by (2 + 1) resonance enhanced multiphoton ionization (REMPI)^19,20^. Another part of the 804 nm (~1.5 mJ) is frequency quadrupled in a device consisting of a tripling unit followed by a sum frequency mixing unit between the third harmonic and the fundamental providing the excitation radiation at ~201 nm with some tunability around this value by fine adjustments of the central wavelength of the amplifier and/or of the quadrupling unit. Resonant radiation is produced for one-photon excitation at the absorption maximum around 201 nm with a full-width-half-maximum (FWHM) of ≈0.3 nm and pulse energies of ~1.2 *μ*J. The horizontal polarization of the pump and probe laser beams is set by means of half-wave plate and the propagation conditions are controlled through adjustable telescopes. Pump and probe beams are propagated collinearly and focused with a 25 cm focal length lens into the vacuum chamber, where they interact with the pulsed molecular beam. The delay between the excitation and detection pulses is controlled by a motorized delay stage placed at the probe laser arm. The instrument temporal response function, considered as the cross correlation of the pump and probe pulses, is measured through multiphoton ionization (MPI) of Xe, obtaining typical values of 150 fs.

The supersonic molecular beam is generated by expansion of the different alkyl iodides at room temperature using helium gas as a carrier gas and a backing pressure of ~1 bar, through a 0.5 mm diameter nozzle of a 1 kHz cantilever piezoelectric pulsed valve^21^. A 1 mm diameter skimmer separates the expansion and ionization chambers, where the molecular beam interacts with the laser pulses.

The ionized iodine fragments are extracted by a set of electrostatic lenses working in velocity mapping configuration^22^ with repeller voltages ~5200 V and optimum conditions found for V_extractor_/V_repeller_ = 0.76 through a field-free time-of-flight (TOF) region (50 cm) until they reach a Chevron configuration dual microchannel plate (MCP), with gated front MCP to achieve mass selection and coupled to a phosphor screen. The images are recorded with a Peltier-cooled 12 bit charge-coupled device camera and later Abel inverted using the polar basis set expansion (pBasex) method^23^. The pixel to energy calibration is performed using the known kinetic energy (KE) release of the CH_3_ (*v* = 0) + I*(^2^*P*_1/2_) and CH_3_ (*v*_1_ = 1) + I*(^2^*P*_1/2_) channels from 201.19 nm photodissociation of CH_3_I^5^. A multidimensional software based on the search for a global minimization employing Levenberg-Marquardt squared residuals minimization algorithm is employed to fit the sequences of images as a sum of contributions characterized by the product of a radial, an angular and a temporal function with adjustable parameters, in order to separate the different contributions that often show partial overlap in one or more dimensions^24^.

### Theoretical method

*Ab initio* multireference configuration interaction (MRCI) electronic structure calculations of the ground and excited potential energy curves including spin–orbit have been performed with MOLPRO (Version 2009.1) for the four linear alkyl iodides^25^. All the calculations were performed in the C_*s*_ symmetry point group, which is common to all the linear alkyls.

Two sets of calculations have been performed employing two different basis sets. A first set was carried out employing the aug-cc-pVDZ of Dunning for the carbon and hydrogen atoms while for iodine, a 46 electrons Dirac-Fock ECP accounting for spin-orbit couplings was used in addition of a basis set for the 7 remaining electrons composed of [2s,3p,2d,1f] with additional diffuse [s,p] functions for a total of [3s,4p,2d,1f]. In the second set, the ANO-TZP basis set^26^ was employed with a supplementary monocentric basis located in the center of charge of the cationic molecule. The center of charge was estimated in the ground state of the cation using the Mulliken charges provided by a previous unrestricted Hartree-Fock (UHF) calculation. This monocentric basis, employed to properly describe the s diffuse orbital, was constructed from even-tempered Gaussian basis with 22 exponents following the progression *α*_n_ = *αβ*^(n−1)^, where *α*=0.01 and *β*=1.46^27^. In order to remove linear dependencies, the basis was contracted to 16 functions.

In the two calculations, the active space contains 6 electrons in 5 orbitals (bond and antibonding for the C–I bond, 2 lone pairs on the I atom and a diffuse s Rydberg orbital). A total number of 13 states were included in the state average, including five ^1^A′, two ^1^A″, four ^3^A′ and two ^3^A″. A regular Douglas-Kroll Hamiltonian^28–30^ and the Atomic Mean Field Interaction (AMFI) approximation were employed^31^ to take into account relativistic effects.

For all molecules, the geometry was optimized and the frequencies were calculated at the single-state CASTP2 level of theory^32,33^ for the ground electronic state using analytical gradients and, thus, the values for the three moments of inertia were obtained. The final 1D potential energy curves, where the radical moiety was frozen at the Franck-Condon geometry, were obtained at MRCI^34^ including the spin-orbit coupling in a perturbative modified frame^35^, *i.e*. the spin-orbit coupling between the electronic states calculated for the different spin multiplicities is evaluated and the resultant matrix is diagonalized, mixing the different multiplicities. Comparable potential energy curves were obtained in both calculations (see Electronic Supplementary Material).

## Results and Discussion

### Iodine fragment translational energy distributions and predissociation lifetimes

**Figure 2:**
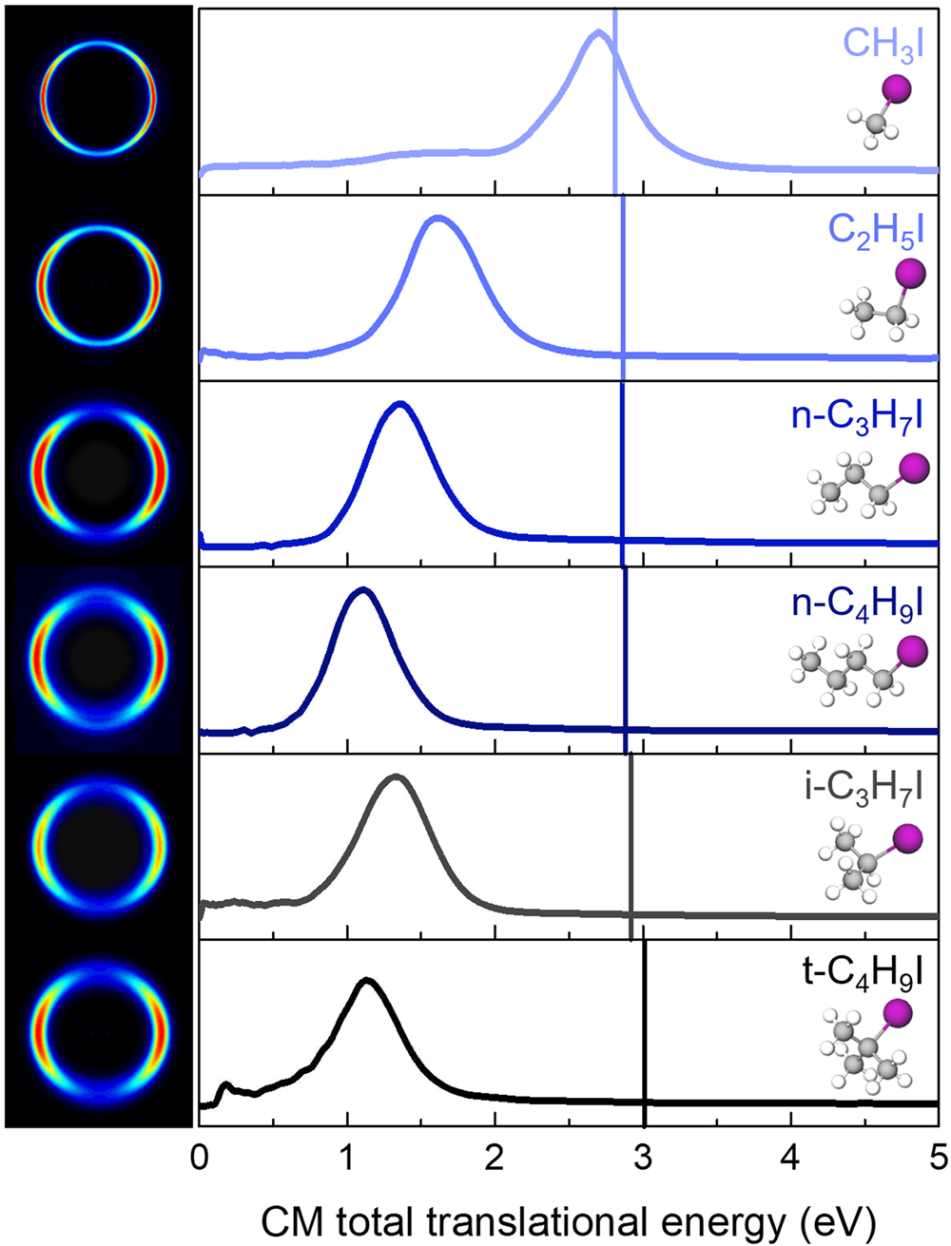
(Left column) Abel-inverted iodine images measured in femtosecond pump-probe VMI experiments for selected linear and branched alkyl iodides following excitation at ~201 nm (pump, see Fig. 1). Both I and I* fragments are detected by (2 + 1) REMPI using a probe laser pulse centered at 304.5 nm. The time delay is fixed at 10 ps. In the images, the radius is proportional to the translational energy of the iodine fragment. The polarization of the pump and probe lasers is vertical. (Right column) Center-of-mass (CM) total translational energy distributions for the different alkyl iodides obtained from the angular integration of the corresponding inverted images are depicted in the right column. The solid vertical lines correspond to the available energy for the I* channel, according to the known dissociation energies of the different molecules indicated in Table 1.

Figure 2 shows a series of Abel-inverted images of the iodine fragment from the photodissociation of all molecules excited at ~201 nm (pump) and detected via (2 + 1) REMPI at 304.5 nm. An asymptotic time delay of 10 ps was selected so that the radical fragment and the iodine atom are considered non-interacting free fragments. Although the REMPI scheme allows the simultaneous detection of both I and I^*^ fragment spin-orbit states, a single perpendicular ring, attributed to the R + I^*^ dissociation channel, is observed in all images becoming broader as the size of the molecule increases. To discard the formation of I, the same images were measured by a (2 + 1) REMPI at 306 nm probe scheme for the exclusive detection of I* and turned out to be identical to the previous ones. Therefore, the quantum yield Φ* defined as [I^*^]/([I^*^] + [I]) does not seem to present significant changes as a function of the size of the molecule, its value being close to unity in all cases^9,20,36,37^. We remark an interesting structure at low radius for *i*-C_3_H_7_I, consisting of parallel and perpendicular sharp rings. They can likely be attributed to some resonant dissociative multiphoton ionization process, which are out of the interest of the present study.

The corresponding total translational energy distributions (TED) obtained from the angular integration of the images are depicted also in Fig. 2. The abscissa axis has been converted from center-of-mass (CM) iodine translational energy to total translational energy by employing the mass factor *m*_RI_/*m*_R_. A single Gaussian-type broad peak is observed for all molecules in agreement with the images. The peak is characterized here by a similar considerable FWHM for all molecules, in contrast with the images. The CM iodine TEDs (not shown here) present indeed a peak which becomes broader with the size of the molecule, accounting for the mass factor. Vertical bars indicate the available energy *E*_*av*_ (summarized in Table 1) for each R + I* dissociation channel given by:

1 $${E}_{av}=h\nu -{D}_{0}-{E}_{{\rm{SO}}}({\rm{I}})+{E}_{i}({\rm{RI}})$$

where *hv* is the excitation photon energy, *D*_0_ the dissociation energy, *E*_SO_(I) is the iodine spin-orbit splitting, 0.943 eV for the I*^20^, and *E*_*i*_(RI) is the internal energy of the parent molecule RI, which is considered negligible in the present work.

**Table 1:** Experimental dissociation energies, *D*_0_, of the alkyl iodides, corresponding available energies for the I* channel, *E*_*av*_, and fraction of the available energy released into internal energy of the radical R, *f*_*int*_, derived from the present experiment (see the text for more details).

	*D*_0_/eV	*E*_*av*_/eV	*f*_*int*_	${{\boldsymbol{\beta }}}_{2}^{{\boldsymbol{init}}}$	${{\boldsymbol{\beta }}}_{2}^{{\boldsymbol{final}}}$	Δ*β*_2_	${{\boldsymbol{\tau }}}_{{{\boldsymbol{\beta }}}_{2}}$/fs	*τ*/fs
CH_3_I	2.41^19^	2.81	0.01	−0.97 ± 0.03	−0.50 ± 0.01	−0.47 ± 0.04	1300 ± 100	1520 ± 100
C_2_H_5_I	2.35^43^	2.87	0.44	−0.89 ± 0.05	−0.59 ± 0.09	−0.30 ± 0.08	1130 ± 151	1341 ± 48
*n*-C_3_H_7_I	2.36^44^	2.86	0.47	−1.08 ± 0.02	−0.74 ± 0.01	−0.34 ± 0.01	677 ± 52	750 ± 91
*n*-C_4_H_9_I	2.34^45^	2.88	0.60	−1.02 ± 0.09	−0.67 ± 0.04	−0.35 ± 0.09	582 ± 78	884 ± 54
*i*-C_3_H_7_I	2.30^44^	2.93	0.56	−0.96 ± 0.06	−0.63 ± 0.03	−0.28 ± 0.05	1625 ± 198	1603 ± 45
*t*-C_4_H_9_I	2.21^45^	3.01	0.61	−0.93 ± 0.03	−0.63 ± 0.02	−0.31 ± 0.05	929 ± 14	1103 ± 72

Uncertainties for the *f*_*int*_ values are around 10%. Experimental values derived from the I^*^ time-resolved images measured at ~201 nm, including the initial anisotropy parameter at early times ${\beta }_{2}^{init}$, the final anisotropy parameter at asymptotic time delays, ${\beta }_{2}^{final}$, the anisotropy variation, Δ*β*_2_, the anisotropy relaxation time_,_ *τ*_*β*_, and the predissociation lifetime*, τ*.

In contrast to CH_3_I, a significant shift between the energy at the maximum intensity of the peak ${E}_{T}^{max}$ and the available energy *E*_*av*_ is observed for all linear and branched alkyl iodides (see Fig. 2), reflecting the internal energy *E*_*int*_ – ro-vibrational – acquired by the co-fragment, since I and I* are atomic species. The fraction of the available energy released into the internal energy of the co-fragment, *f*_*int*_, is derived from:

2 $${f}_{{int}}=\frac{{E}_{{int}}}{{E}_{{av}}}=\frac{{E}_{{av}}-{E}_{T}^{{\max }}}{{E}_{{av}}}$$

The *f*_*int*_ values summarized in Table 1 highlight a clear trend. As the size of the linear radical R increases, and thus the vibrational degrees of freedom, a larger fraction of *E*_*av*_ is released into internal energy in spite of translational motion. We remark that the branched *i*-C_3_H_7_I presents a remarkable higher *f*_*int*_ compared to the linear *n*-C_3_H_7_I, in contrast to *n*-C_4_H_9_I and *t*-C_4_H_9_I, characterized by a similar *f*_*int*_.

**Figure 3:**
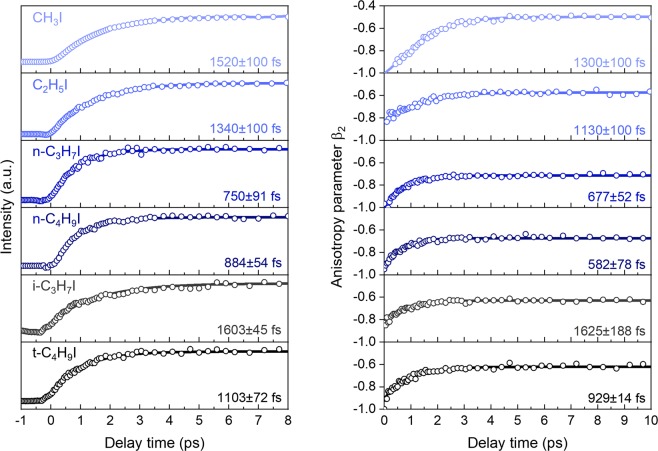
Left panel: Experimental transients corresponding to the I* atom appearance after *B*-band excitation at ~201 nm and detected through (2 + 1) REMPI scheme at 304.5 nm for the studied alkyl iodides. The lifetimes of the excited states are indicated in the corresponding panel and summarized in Table 1. Right panel: Evolution of the anisotropy parameter, *β*_2_ (open circles), resulting from the fitting of the measured angular distributions for I* respect to the laser polarization direction to Eq. (4), as a function of the pump-probe time delay. Solid lines result from the fitting to Eq. (5).

The evolution of the intensity of the iodine fragment images as a function of the pump-probe time delay has been monitored in order to *clock* the reaction time, *i.e*. measuring the C-I bond cleavage times and, in particular, the predissociation lifetime in the present case. The main contribution corresponding to the ring in Fig. 2 is isolated through the use of a multidimensional fitting procedure^24^ and integrated as a function of time to obtain the transients shown in Fig. 3. The transients are fitted to a rising exponential function convoluted with the instrumental response function using the following equation:

3 $$S(t)=A{e}^{-4{\rm{l}}{\rm{n}}2{\left(\frac{t}{{\tau }_{cc}}\right)}^{2}}\otimes [(1-{e}^{-\frac{t-{t}_{0}}{\tau }})H(t-{t}_{0})]$$

where *τ* is the predissociation lifetime, *τ*_*cc*_ is the instrumental response function time, *t*_0_ is the time of pump-probe temporal overlap, *H*(*t*) is the Heaviside step function, and *A* is an amplitude factor. The lifetimes *τ* derived from this method are summarized in Table 1. The obtained lifetime decreases considerably as the size of the linear molecule increases, leveling off for butyl iodide. In contrast, lifetimes are considerably longer for the two branched molecules although diminishing significantly for *t*-C_4_H_9_I compared to *i*-C_3_H_7_I.

### Time-resolved iodine atom anisotropy and parent rotational temperature

In order to analyze in detail the evolution of the anisotropy during predissociation, angular distributions have been obtained by radial integration of the inverted images at each time delay. The corresponding anisotropy parameters are obtained by fitting the angular distribution to^38^:

4 $$I(\theta )=\frac{\sigma }{4\pi }[1+{\beta }_{2}{P}_{2}(\cos \,\theta )]$$

where *σ* is the total absorption cross section, *θ* is the angle between the polarization axis of the photolysis laser and the fragment velocity vector and *P*_2_(cos*θ*) is a second order Legendre polynomial. As the experimental setup has not been calibrated for absolute intensities, the term $\frac{\sigma }{4\pi }$ is nevertheless considered as a normalization fitting parameter. Since I* presents an angular momentum *J* = 1/2, it cannot show photofragment polarization effects, and a single *β*_2_ anisotropy parameter is used to fit the angular distributions according to Eq. (4). Therefore, *β*_2_ coincides with the photodissociation anisotropy parameter *β*.

Likewise, the temporal evolution of the anisotropy parameter over time, *β*_2_(*t*), depicted in Fig. 3 for all the molecules studied, can be described by:

5 $${\beta }_{2}(t)={\beta }_{2}^{init}+\Delta {\beta }_{2}{e}^{-\frac{t-{t}_{0}}{{\tau }_{{\beta }_{2}}}}$$

where ${\beta }_{2}^{init}$ represents the initial value of the anisotropy parameter (at time delay zero), Δ*β*_2_ is the total variation of *β*_2_ over time, and ${\tau }_{{\beta }_{2}}$ is the time constant of the exponential rise of the anisotropy over time. The initial ${\beta }_{2}^{init}$ and final ${\beta }_{2}^{final}$ (at asymptotic time delay of ~10 ps) values obtained are listed in Table 1 along with the Δ*β*_2_ and ${\tau }_{{\beta }_{2}}$ values.

For all studied alkyl iodides, the ${\beta }_{2}^{init}$ show negative values close to −1, reflecting the main contribution of a perpendicular transition, *i.e*. a transition dipole moment perpendicular to the C-I bond, consistent with the initial population of the [6*A*″, 7*A*′] Rydberg states. However, *β*_2_ decreases considerably with time delay, leading to final values ranging between ~−0.7 and −0.5 depending on the molecule. For the linear molecules, the loss of anisotropy seems to decrease as the size of the molecule increases, while the branched molecules are characterized by a slightly lower ${\beta }_{2}^{init}$ and ${\beta }_{2}^{final}$ compared to their respective linear ones.

The remarkable loss of anisotropy is attributed to the rotation of the parent molecule during the excited state lifetime^5–7,9,15^, since the predissociation time *τ* is similar to the rotational period of the molecule *τ*_*rot*_. Therefore, it can be directly related to the initial rotation of the parent molecule, *i.e*. the rotational temperature in the molecular beam. Following classical models^39,40^, the final anisotropy parameter, *β*^*final*^, for a one-photon perpendicular transition can then be described as:

6 $${\beta }^{final}(\omega ,\tau )={\beta }^{init}\frac{1+{(\omega \tau )}^{2}}{1+4{(\omega \tau )}^{2}}$$

where *β*^*init*^ = −1 for a perpendicular transition and *ω* is the modulus of the angular velocity of the excited molecule such that the rotational energy is ${E}_{rot}=({I}_{a}{\omega }_{a}^{2}+{I}_{b}{\omega }_{b}^{2}+{I}_{c}{\omega }_{c}^{2})/2$, and *I*_*a*_, *I*_*b*_ and *I*_*c*_ are the three moments of inertia of the molecule.

Besides, the product angular distribution can be written as:

7 $$I(\theta )={\int }_{0}^{\infty }B(\omega ;{T}_{rot})\frac{\sigma }{4\pi }[1+{\beta }^{final}(\omega ,\tau ){P}_{2}(\cos \,\theta )]d\omega $$

where *B*(*ω*; *T*_*rot*_) is the Boltzmann distribution for the rotational temperature *T*_*rot*_ of the parent molecule, and can be expressed as

8 $$B(\omega ;{T}_{rot})={e}^{-\frac{{E}_{rot}}{{k}_{B}{T}_{rot}}}={e}^{-\frac{({I}_{a}{\omega }_{a}^{2}+{I}_{b}{\omega }_{b}^{2}+{I}_{c}{\omega }_{c}^{2})}{2{k}_{B}{T}_{rot}}}$$

where *k*_*B*_ is the Boltzmann constant.

The moments of inertia have been computed for all the molecules studied and are summarized in Table 2. Methyl iodide is a prolate symmetric top and is characterized by having three main non-null moments of inertia, two of them being equal (*I*_*b*_ = *I*_*c*_) and much larger than the third one (*I*_*a*_). By approximation to a diatomic molecule, a single moment of inertia *I*_*b*_, oriented along the carbon-iodine bond was considered in previous works^7,9^. The classical rotational energy distribution was then calculated and *T*_*rot*_ was directly deduced using Eq. (7). A similar approximation was also recently considered for ethyl iodide^15^.

**Table 2:** Calculated moments of inertia (*I*_*a*_, *I*_*b*_ and *I*_*c*_) for the molecules considered in this work.

	*I*_*a*_/amu·Å^2^	*I*_*b*_/amu·Å^2^	*I*_*c*_/amu·Å^2^	*τ*/fs	*T*_*rot*_/K
CH_3_I	3.30	68.31	68.31	1520 ± 100	${54}_{-3}^{+4}$
C_2_H_5_I	17.41	172.61	183.56	1341 ± 48	${25}_{-0}^{+1}$
*n*-C_3_H_7_I	20.49	393.09	403.95	750 ± 91	${40}_{-4}^{+6}$
*n*-C_4_H_9_I	33.41	700.26	720.88	884 ± 54	${39}_{-7}^{+2}$
*i*-C_3_H_7_I	63.37	235.06	282.10	1603 ± 46	${20}_{-0}^{+1}$
*t*-C_4_H_9_I	112.22	328.55	328.55	1103 ± 72	${27}_{-2}^{+1}$

Rotational temperatures (*T*_*rot*_) are estimated with the model described by Eq. (7) (see text) taking into account the experimental predissociation lifetimes (*τ*) and their error bars. The upper and lower error bars of T_rot_ are obtained when considering the upper and lower values of the predissociation lifetimes *τ*.

Due to the large mass of the iodine atom, it is, however, relatively easy to populate rotational levels associated to small moments of inertia, even at low temperatures. Besides, as observed in Table 2, the moments of inertia increase considerably with the size of the molecule, in particular for the two branched molecules, where the tree moments are almost of the same order of magnitude. This will also contribute to an easier population of rotational levels. The approximation to a diatomic molecule is then no longer valid beyond the case of CH_3_I. In the present work, the three moments of inertia are taken into account in Eq. (8) following the model described by Eq. (7). Since Eq. (8) cannot be easily calculated classically when all three moments of inertia have a role, it is more convenient to consider the Boltzmann distribution over the quantum rotational levels. Therefore, all rotational levels up to *J*_*rot*_ = 500 have been computed for all molecules, using the CALPGM program^41,42^. The Boltzmann distribution resolving Eq. (8) is then straightforward, and Eq. (7) can be easily solved for a considered temperature.

An iterative procedure has then been applied consisting of simulating the final angular distribution considering the experimental values of *β*^*init*^, *β*^*final*^ and *τ*. The normalized angular distribution has been simulated by resolving Eq. (7) for temperatures between 0 and 300 K in steps of 1 K until it converges to the experimental angular distribution. The rotational temperatures has been derived and are summarized in Table 2. The estimated error bars of the measured lifetimes have been considered and the subsequent non-negligible variation in *T*_*rot*_ is also indicated in Table 2 with upper and lower error bars.

### Predissociation mechanism and structural effects

In order to explain the variation of the predissociation lifetimes for the series of linear alkyl iodides, which implies reductions of 12%, 51% and 42%, respectively, for the series of ethyl, *n*-propyl and *n*-buthyl iodides, with respect to the lifetime of CH_3_I, and to give insight on the predissociation mechanism and the associated structural effects, we have calculated the *ab initio* potential energy curves (PECs) as a function of the C-I distance for the four linear alkyl iodides including all Rydberg states up to 8 eV, using the same level of theory and two different basis sets. All the PECs are depicted in Fig. S1. Associated vertical excitation wavelengths to the *B*-band have been estimated from the minimum of the interpolated PECs and are reported in Table S1. As observed, a pretty good agreement with the experimental excitation wavelengths is found. The PECs for the linear series of alkyl iodides show remarkable similarities; likewise the experiments should reflect a comparable reaction mechanism leading to the formation of R + I^*^. Several repulsive states associated with the first absorption *A*-band are observed as well as the different Rydberg states characterizing the *B*- and *C*-bands. In addition, the excited state corresponding to the ion pair formation, presenting a minimum at longer C-I distances around 3.5 Å, appears to be stabilized for an increasing size of the molecule and the repulsive states 4*A*″ and 5*A*′, which correspond to the ^1^*Q*_1_ in CH_3_I, increasingly cross the Rydberg states comprising the *B*-band as the size of R increases.

Following excitation at ~201 nm, *i.e*. the ${0}_{0}^{0}$ transition for the series of linear molecules, the initially populated bound Rydberg states 6*A*″ and 7*A*′ (the doubly degenerate ^3^*R*_1_ in CH_3_I) can undergo predissociation through the observed crossing with the purely repulsive states 7*A*″, 8*A*′ and 8*A*″ (the ^3^*A*_1_(*E*) and ^3^*A*_1_(*A*_2_) states in CH_3_I), leading to the major formation of R + I^*^, in agreement with the experimental results. Based on the present theoretical results showing the absence of any competing mechanisms, the measured predissociation lifetimes ranging 0.75–1.5 ps must therefore reflect the dynamics around the crossing: either the position of the curve crossing with respect to the Franck-Condon region or the diabatic coupling between the Rydberg and repulsive states.

**Figure 4:**
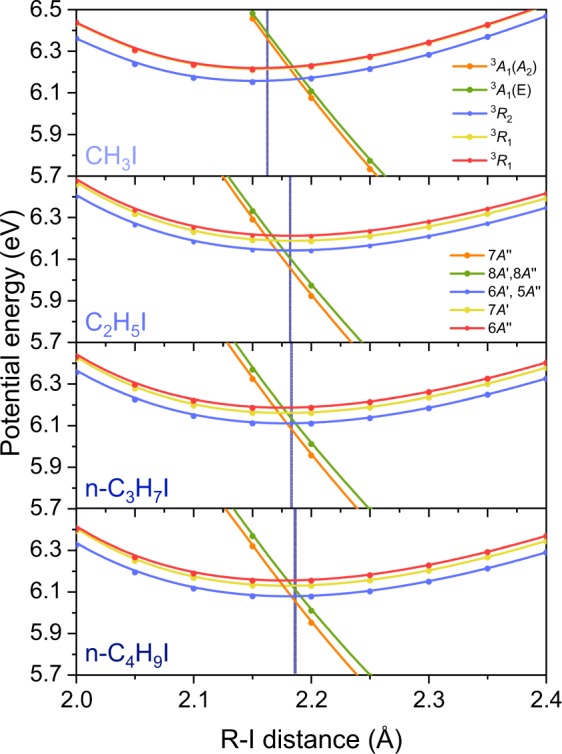
Expanded view of the potential energy curves for the linear alkyl iodides, CH_3_I, C_2_H_5_I, *n*-C_3_H_7_I and *n*-C_4_H_9_I, taken from the top panels of Fig. S1, showing the initially populated 6*A*″ and 7*A*′ Rydberg states (^3^*R*_1_ in CH_3_I) as well as the curve-crossing region leading to electronic predissociation. Vertical bars indicate the C-I equilibrium distance in the ground state for each molecule. We have made the adiabatic curves to cross for a better visualization of the subtle changes in the crossing region in going from one molecule to the next with increasing structural complexity. No diabatization was performed though. Interestingly, the doubly degenerate ^3^*R*_1_ Rydberg states in CH_3_I, split into to non-degenerate 6*A*″ and 7*A*′ states for the rest of linear alkyl iodides.

Figure 4 depicts expanded views of the adiabatic PECs for the series of linear molecules shown in the top panels of Fig. S1. In these plots, the curves have been made to cross to facilitate the view of the crossing region, but no diabatization has been carried out. A version of these plots without crossing the curves can be found in Fig. S2 of the supplementary material. In both Figures, the vertical line in each plot represents the center of the Franck-Condon region from the minimum of the ground state potential energy curve. As observed, the crossing is located to the right of the vertical bar for CH_3_I, whereas the opposite situation occurs for the larger linear alkyl iodides, *i.e*. the crossing is to the left of the vertical bars. In addition, the crossing gets slightly closer to the ground state C-I equilibrium distance for an increasing size of the linear molecule. This would suggest that the closer the crossing is to the Franck-Condon region, the more favorable is the transfer of population from the initially populated Rydberg states into the repulsive states, leading therefore to the shorter lifetimes measured for an increasing size of the linear chain. Such shift is nevertheless small and, in addition, the computed coupling between the Rydberg and the repulsive states appears to be similar for all molecules. Therefore, additional structural effects on the coupling between the states must occur.

The PECs have been computed in a single dimension, along the C-I reaction coordinate. For an increasing size of the radical, a second or even more coordinates could enhance the coupling, leading to a reduction of the lifetime. This means that in the normal mode coordinates, a specific vibrational mode could induce dynamical effects. Moreover, the role of a vibrational mode on the coupling between excited states generally entails some further vibrational excitation of the molecular fragment produced. The increasing *f*_*int*_ for the larger molecules could this way reflect a greater effect of a particular vibrational mode on the coupling leading to predissociation. Alekseyev *et al*.^8^ already predicted an enhancement of the non-adiabatic coupling by means of the *v*_6_ rocking vibration mode in methyl iodide, while experiments performed exciting the ${2}_{0}^{1}$ vibronic band demonstrated that the *v*_2_ umbrella mode yielded faster predissociation lifetimes^9^. Some umbrella or bending vibrational mode could similarly enhance the non-adiabatic coupling in the other linear alkyl iodides, leading to faster predissociation. Further time-resolved VMI experiments exciting selected vibronic transitions so that the amplitude of a particular mode is increased would be particularly valuable in combination with *ab initio* calculations in selected dimensions.

We note that for the two branched alkyl iodides, *i*-C_3_H_7_I and *t*-C_4_H_9_I, the excitation wavelengths corresponding to the origin of the *B*-band (${0}_{0}^{0}$ transition), 201.81 nm and 203.75 nm, respectively^17^, could not be selected due to the limited tunability of our femtosecond laser system around 201.0 nm. Therefore, for the branched *i*-C_3_H_7_I, absorption with the selected wavelength (201.06 nm) is tentatively attributed to the vibronic band associated with the C-I stretching mode^17^, while for *t*-C_4_H_9_I, the excitation wavelength of 201.18 nm is assigned to absorption to the ${6}_{0}^{1}$ vibronic transition of the *B*-state^18^. There are 4 vibrational modes active in this molecule, of which the *v*_6_ corresponds to the C-C stretching mode (806 cm^−1^ in the ground state). The lifetimes measured for these two molecules at the selected vibronic states are longer (1603 ± 45 fs and 1103 ± 72 fs) than the corresponding lifetimes of their linear counterparts (750 ± 91 fs and 884 ± 54 fs) by factors of 2.14 and 1.25, respectively^15^. In general terms, vibronic excitation in CH_3_I and C_2_H_5_I yields shorter lifetimes than those measured for the ${0}_{0}^{0}$ transitions. For instance, the lifetimes of the ${0}_{0}^{0}$ vibronic bands for CH_3_I and C_2_H_5_I are, respectively, 1520 ± 100 fs and 1341 ± 48 fs, whereas those lifetimes for the ${2}_{0}^{1}$ and ${18}_{0}^{1}$ vibronic bands for the two molecules are, respectively, 0.86 ± 0.04 ps and 0.94 ± 0.03 ps. The fact that in the branched molecules we are measuring predissociation lifetimes after vibronic excitation, which are longer that those lifetimes of the linear counterparts, seems to indicate that the crossing region is affected by the complexity of the branched molecules in a way that is intermediate to that of the corresponding linear molecules. *Ab initio* calculations as those carried out for the linear alkyl iodides would be very timely for the branched alkyl iodides to understand the measured lifetimes. Therefore, besides some structural effect on the non-adiabatic coupling between the Rydberg and the repulsive states, the remarkably larger lifetimes measured could be directly related to the initially populated vibronic state.

A second dissociation channel R + I, corresponding to the formation of I in its ground spin-orbit state was reported for methyl iodide and attributed to a second curve crossing between the initially populated 6*A*″ and 7*A*′ Rydberg states and the 4*A*″ and 5*A*′ repulsive states (corresponding to ^1^*Q*_1_) from the *A*-band, located at shorter C-I distances. For an increasing size of the linear radical, the repulsive 4*A*″ and 5*A*′ are pushed inside the Rydberg states, as observed in Fig. S1. This would suggest a more favorable formation of I for an increasing structural complexity, in agreement with our previous investigation on the predissociation dynamics of ethyl iodide employing nanosecond lasers^14^. However, in the present experiment, despite all the efforts, the I channel was not detected following excitation of the selected molecules and this would lead to the conclusion that the I channel remains a very minor reaction pathway. The coupling between those states and/or the overlap with the vibrational ground state wavefunction must therefore be rather unfavorable, regardless of the increasing structural complexity and higher degrees of freedom of the molecule. It is possible, however, that if the trend follows, the I channel can become a competing channel for more complex alkyl iodides.

## Conclusions

In this work, we have evaluated the correlation between chemical structure and the predissociation dynamics following excitation on the second absorption band (*B*-band) at ~201 nm of a series of linear and branched alkyl iodides with increasing structural complexity. Femtosecond time-resolved velocity map ion imaging experiments employing resonance enhanced multiphoton ionization (REMPI) for the detection of iodine fragments have been performed along with high-level *ab initio* calculations of potential energy curves as a function of the C-I distance. The electronic predissociation lifetimes ranging between 0.8 and 1.5 ps were derived from the measured iodine atom transients, while the temporal evolution of the anisotropy was analyzed and discussed. In agreement with the results, for all molecules, the initially populated bound Rydberg states 6*A*″ and 7*A*′ undergo major predissociation through a crossing with the purely repulsive states 7*A*″, 8*A*′ and 8*A*″ leading to the major R + I^*^ channel. The I channel, formed through a second crossing with the 4*A*″ and 5*A*′ repulsive states, is found to remain a minor channel and could not be observed in the present experiments. The reported lifetimes are found to decrease for an increasing size of the linear molecules, reflecting the small shift observed in the position of the crossing of the potential energy curves involved, and very likely a greater non-adiabatic coupling between the initially populated Rydberg states and the repulsive states leading to dissociation induced by increasing additional vibrational mode coordinates as the size of the radical increases. The loss of anisotropy appears to be fully accounted for by the parent molecular rotation during predissociation and thus the rotational temperature of the parent molecule in the molecular beam can be derived.

## Supplementary information

Supplementary Information.
