# Supplementary material for: Structural dynamics effects on the electronic predissociation of alkyl iodides
Source: Sci Rep. 2020 Apr 21;10:6700. doi: 10.1038/s41598-020-62982-0 (PMC7174404; doi:10.1038/s41598-020-62982-0)
Supplement: Supplementary file 1 — Supplementary Information. [file 41598_2020_62982_MOESM1_ESM.pdf]

# Structural dynamics effects on the electronic predissociation of alkyl iodides

**M.L. Murillo-Sánchez, A. Zanchet, S. Marggi Poullain, J. Gonzalez-Vázquez, L. Bañares**

## ABSTRACT

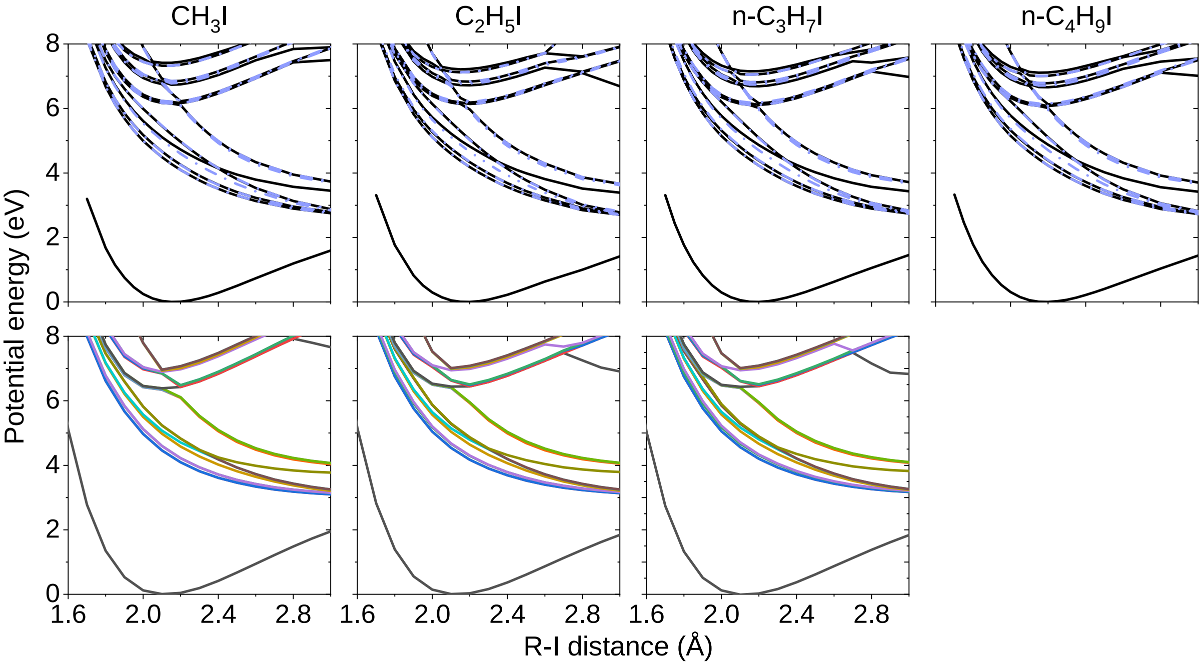


**Figure S1.** MRCI adiabatic potential energy curves as a function of the C-I distance for the linear alkyl iodides. Two sets of calculations have been performed employing two different basis sets. A first set (top panels) was carried out employing the aug-cc-pVDZ of Dunning for the carbon and hydrogen atoms while for iodine, a 46 electrons Dirac-Fock ECP accounting for spin-orbit couplings was used in addition of a basis set for the 7 remaining electrons composed of [2s,3p,2d,1f] with additional diffuse [s,p] functions for a total of [3s,4p,2d,1f]. In the second set (bottom panels), the ANO-TZP basis set was employed with a supplementary monocentric basis located in the center of charge of the cationic molecule. The center of charge was estimated in the ground state of the cation using the Mulliken charges provided by a previous unrestricted Hartree-Fock (UHF) calculation. Further details of each calculation can be found in the theoretical methodology. Common features detailed in the text are distinguished at both theory levels. In the top panels, solid lines refer to states of $A'$ symmetry in the $C_s$ point group and the dashed lines are for $A''$ states. In the bottom panels, the colors represent the states as in Ref.[1] for ethyl iodide.

**Table S1.** Comparison of the experimental and theoretical excitation wavelengths for the studied linear ($CH_3I$, $C_2H_5I$, $n$-$C_3H_7I$, $n$-$C_4H_9I$) alkyl iodides. Experimental values are taken from Refs.[2,3].

| Molecule | Experimental wavelength (nm) | Theoretical vertical wavelength (nm) |
|---|---|---|
| $CH_3I$ | 201.19 | 199.6 |
| $C_2H_5I$ | 201.19 | 200.4 |
| $n$-$C_3H_7I$ | 201.12 | 201.2 |
| $n$-$C_4H_9I$ | 201.18 | 202.2 |

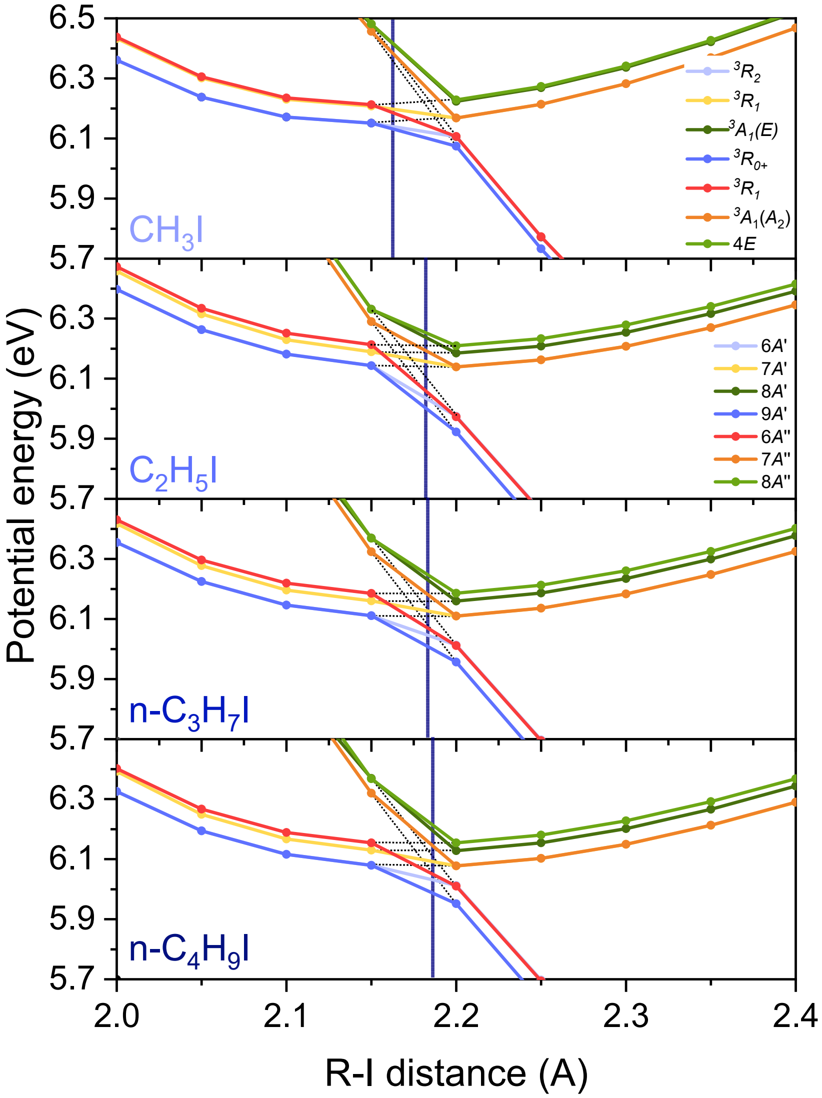


**Figure S2.** Same as in Figure S1 but in expanded view to better appreciate the details of the crossing region in the second absorption *B*-band. Dashed black lines represent the extrapolation of the different curves to better appreciate the crossings between the Rydberg states and the purely repulsive states. The vertical blue line represents the vertical excitation at the Franck-Condon geometry from the ground state. The electronic symmetry of relevant states is specified within the Franck-Condon geometry. In the case of methyl iodide, the crossing remains at longer bond distances than the excitation region; whereas in crescent-chain alkyl iodides, this crossing is located at shorter bond distances than the excitation region; although the crossing is mostly located over the Franck Condon region.

## References

1. Marggi Poullain, S. *et al.* Dynamics of the photodissociation of ethyl iodide from the origin of the B band. a slice imaging study. *Phys. Chem. Chem. Phys.* **21**, 14250–14260, DOI: 10.1039/C8CP06482B (2019).

2. Boschi, R. & Salahub, D. The far ultra-violet spectra of some 1-iodoalkanes. *Mol. Phys.* **24**, 289–299, DOI: 10.1080/00268977200101451 (1972).

3. Boschi, R. & Salahub, D. The far ultra-violet spectra of some branched chain iodo-alkanes, iodo-cyclo-alkanes, fluoro-iodo-alkanes and iodo-alkenes. *Mol. Phys.* **24**, 735–752 (1972).
